# The effects of locomotion on bone marrow mesenchymal stem cell fate: insight into mechanical regulation and bone formation

**DOI:** 10.1186/s13578-021-00601-9

**Published:** 2021-05-17

**Authors:** Yuanxiu Sun, Yu Yuan, Wei Wu, Le Lei, Lingli Zhang

**Affiliations:** 1 School of Physical Education & Sports Science, South China Normal University, 55 Zhongshan Road West, Tianhe District, Guangzhou, 510631 Guangdong China; 2 Department of Orthopaedics, The Second Affiliated Hospital and Yuying Children’s Hospital of Wenzhou Medical University, Wenzhou, 325000 Zhejiang China; 3 School of Sport and Health, Guangzhou Sport University, Guangzhou, 510500 Guangdong China; 4 School of Kinesiology, Shanghai University of Sport, Shanghai, 200438 China

**Keywords:** Exercise, Mechanoresponse, Signaling pathway, Differentiation

## Abstract

Bone marrow mesenchymal stem cells (BMSCs) refer to a heterogeneous population of cells with the capacity for self-renewal. BMSCs have multi-directional differentiation potential and can differentiate into chondrocytes, osteoblasts, and adipocytes under specific microenvironment or mechanical regulation. The activities of BMSCs are closely related to bone quality. Previous studies have shown that BMSCs and their lineage-differentiated progeny (for example, osteoblasts), and osteocytes are mechanosensitive in bone. Thus, a goal of this review is to discuss how these ubiquious signals arising from mechanical stimulation are perceived by BMSCs and then how the cells respond to them. Studies in recent years reported a significant effect of locomotion on the migration, proliferation and differentiation of BMSCs, thus, contributing to our bone mass. This regulation is realized by the various intersecting signaling pathways including RhoA/Rock, IFG, BMP and Wnt signalling. The mechanoresponse of BMSCs also provides guidance for maintaining bone health by taking appropriate exercises. This review will summarize the regulatory effects of locomotion/mechanical loading on BMSCs activities. Besides, a number of signalling pathways govern MSC fate towards osteogenic or adipocytic differentiation will be discussed. The understanding of mechanoresponse of BMSCs makes the foundation for translational medicine.

## Introduction

Bone is the largest loading-bearing organ in our body, which provides mechanical integrity for locomotion and protection. During the whole life span, bone tissue undergoes constant bone damage and remodelling to contribute to bone homeostasis [1, 2]. Bone mass is regulated by bone resorption and bone formation. In the pre-developmental stage and development period, bone formation rate is quicker than the rate of bone resorption, and bone grows rapidly. In the adult stage, the bone resorption and bone formation reach an equilibrium state, thereby stabilizing the bone mass. The bone resorption is greater than bone formation in postmenopausal women, leading to bone loss and osteoporosis. The existence and structure of bone depends on the formation, quantity, and activity of bone cells that are regulated by several factors, such as mechanical and chemical stimuli, receptor and signal transduction, transcription and translation, heredity, nutrition, and endocrines. The dynamic equilibrium between the damage and remodelling largely relies on regular exercises as physical activities provide mechanical stimulus to initiate various bone cell activities to maintain bone homeostasis [3, 4].

Recent studies have greatly expanded the knowledge of mechanoresponse of mesenchymal stem cells and their roles on the maintenance of bone homeostasis [5]. Previous studies have shown that bone marrow mesenchymal stem cells (BMSCs) and their lineage-differentiated progeny (for example, osteoblasts), and osteocytes are mechanosensitive in bone. Many studies of BMSC mechanosensibility have indicated central roles of BMSCs in locomotion induced bone mass increase [6–8]. As a type of multipotential cells, BMSCs can differentiate into chondrocytes, osteoblasts, and adipocytes under different loading condition [9]. And it is suggested that constant loading in an appropriate intensity is able to switch the differentiation direction of BMSCs from adipogenesis to osteoblastogenesis [7, 8]. Besides, the increase of BMSCs proliferation induced by fluid shear stress (FSS) also contributes to the number of total bone cell population [10]. Accordingly, a direct connection between locomotion and enhancement of bone quality is built in light of the mechanical ability of BMSCs.

The differentiation of MSC towards an adipogenic or osteogenic lineage relies on various of signalling pathways and transcription factors. The most well-known key factors for adipogenic or osteogenic cell fate decision are peroxisome proliferator-activated receptor γ (PPARγ), the master regulator for adipogenesis, and runt related transcription factor 2 (Runx2), the main regulator for osteogenesis [11, 12]. These two transcription factors are downstream to several signalling pathways including: bone morphogenetic protein (BMP), insulin-like growth factor (IGF), notch, hedgehog (Hh) and wnt signalling [13]. Interestingly, many of these signalling pathways are dual effectors for both adipogenesis and osteoblastogenesis [13]. For example, while IGF-1 and its receptor could promote proliferation and differentiation of adipocyte progenitors [14], they also show a significant potential in bone formation [15, 16]. Therefore, understanding of how mechanical cues regulate the signalling pathways in BMSCs is of significance, by which the mystery of the effects of locomotion on bone formation could be unlocked. This review will discuss the effects of locomotion on BMSCs activities and the variation of signalling pathways in loading conditions.

## The mechanical environment of BMSCs

Considering the ubiquity of BMSCs, there is no such a classical mechanoenvironment that describes the accommodation of BMSCs. Current studies majorly focused on the mechanical environment of bone marrow, as it houses the largest percentage of total BMSC population [17]. To narrow the focus, the region between marrow tissue and blood-vessels walls are particularly important in the analysis of loading-driven activities of BMSCs, since most of BMSCs are in fact perivascular cells [18]. The local hydrostatic pressure, shear stress substrate strains and topography [3, 19] are combined to create the mechanical environment of BMSCs.

A study regarding to extensive necrosis of the bone firstly caught public attention on the effects of intramedullary pressure (IMP) [20]. Numerous subsequent studies established the relationship between systemic blood pressure (110–140 mmHg) and IMP (30 mmHg), which was then described to obey the one-fourth rule [21]. Normally, medullary pressure remains essentially constant as long as the arterial pressure is above 81 mmHg [22]. In this condition, BMSCs could maintain the stemness of hematopoietic stem/progenitor cell through their adherence, thus, contribute to local homeostasis [23]. However, the stabilization of IMP can easily be destroyed when external factors are involved such as occlusion of regional vessels, injection of epinephrine, norepinephrine, acetylcholine, pressor and depressor drugs as well as skeletal muscle contraction [17]. The change of IMP could certainly influence the mechanoresponse of BMSCs.

On the other hand, the response of BMSCs to their mechanical environment also depends on fluid shear stress from bone matrix. However, the rheology properties of bone marrow make the in vitro simulation of BMSC mechanical environment very difficult, which leaves us with the question concerning what forms of mechanical cues BMSCs experience in local environment [3]. So far, different modes of mechanical stimulus, such as compression, mechanical stretch and fluid flow shear stress, have been applied to BMSCs in vitro, and an increasing number of studies have concluded that the reaction of BMSCs are not only mode, but also intensity and duration dependent [24]. For example, increased proliferation of MSCs was observed with the short-term stimulation of oscillatory fluid flow (OFF) [10], while 3-h constant stimulation with similar stress could cause osteogenic differentiation of BMSCs [25]. Accordingly, it is reasonable to assume that external stress induced variation of bone marrow mechanoenvironment could trigger various activities of BMSCs.

Another factor related to BMSC mechanoresponse is the stiffness of extracellular matrix (ECM). “Stiffness” is termed as a metric of the rigidity which is sensed by cells via application of cell‑generated forces [19]. Much stiffer as the bone marrow is than other ECM, a higher force is required for the deformation of its network [19]. Correspondingly, the deformation of bone marrow would create higher tractions transmitted to cells through integrin-mediated cell-ECM interactions [26]. A previous study has reported computational prediction of fluid flow stress in the interface of bone marrow was approximately 50 dyn/cm^2^, which is far higher than the threshold to activate BMSCs in vitro [27]. However, whether BMSCs could sense this stress and how they react to such intensity remains largely unknown.

## Mechanobiology of BMSCs

### ECM-to-BMSC mechanotransduction

The complexity of BMSC mechanosensation comes from the mechanism of how mechanical cues are transmitted and terminally transferred into molecular signals which result in various cellular activities. To answer these questions, several studies have investigated the cell-ECM and cell–cell adhesion as the major structures for mechanical sensation [28]. Integrin, as a membrane protein, is the bridge between ECM and intracellular compartments in mechanical transmission [29–31]. It is clustered into multiprotein complexes called focal adhesion to mechanically integrate extracellular and intracellular compartments [28]. The mechanical sensibility of integrin is reflected on its conformational changes under mechanical stimulation [32]. When sensing the mechanical cues, integrin reshapes its ectodomain to facilitate the formation of adhesion structures bridging the cell membrane to cytoskeleton [29]. In this way, focal adhesion could assemble the actin bundles, therefore, generate the tension into cells [33]. During this process, actomyosin cytoskeleton is the key structure which provides the driving force to generate the tension which is regulated by Rho family of GTPases [30]. Recent studies have revealed that actomyosin cytoskeleton is predominant regulator of YAP (Yes-associated protein) and TAZ (transcriptional co-activator with PDZ-binding motif). These two transcriptional regulators could sense a broad range of mechanical cues and generate mechanical signals from ECM to intracellular matrix [34], including ECM rigidity and topology, stretching and tension force. The disruption of mechanical transmission mediated by YAP and TAZ can be observed both in the condition of F-actin depolymerization and RHO inhibition [35–37]. This could lead to severe defect of BMSC proliferation, since contact inhibition of cell proliferation has been proved to relate to the inactivation and phosphorylation of YAP and TAZ [38].

Moreover, in recent years, it has been found that Piezo1, a mechanosensitive ion channel protein, can transfer mechanical signals through deformation to the pore nucleus structure of its protein, thus opening channels and mediating ion into cells, regulating the transcription of downstream genes. At the mechanistic level, in response to mechanical loads, Piezo1 in osteoblastic cells controls the YAP-dependent expression of type II and IX collagens. In turn, these collagen isoforms regulate osteoclast differentiation [39].

Taken together, the findings of integrin, actin bundles and their correlation to the downstream molecules like YAP and TAZ unlocked the previously unanswered questions about ECM-to-BMSC mechanotransmission.

### Role of cilium in mechanical sensation and transduction

Primary cilia is a microtubule-based antenna-like sensory organelles critical for mechanical transduction. transducing extracellular mechanical and chemical signaling [40]. Motor cilium perform various biological functions through their beating movement, including mixing fluids and transporting food particles. Non-motor cilium act as sensors and send signals to the cell about its microenvironment [41]. Shear stress caused by fluid flow could have a certain effect on cell metabolism through cilium. The role of primary cilium in integrating shear stress and maintaining the execution of specific cellular procedures has been confirmed [42]. The structure of cilium is based on axons, which are cylindrical arrays of microtubules, directly templated by the mother center of the centrosome (usually called the matrix) [43]. Cilium can be classified according to their different microtubule arrangements and whether there is movement. The primary cilium is a solitary microtubule-based organelle that grows from the mother centriole and projects from the cell surface in many vertebrate tissues, including bone, nervous system, carcinoma, kidney, cartilage and cardiovascular tissues [44]. The neurons of mammalian brain have primary cilium, which are rich in a series of G protein-coupled receptors (GPCRs). Through melanin-concentrating hormone receptor 1 (MCHR1), the length of cilium can be changed to regulate neuronal activity and physiological functions, so as to achieve feeding and memory tasks [45].

In the process of fracture healing, primary cilium act as sensory organelles that mediate several signalling pathways [46]. Microtubules form the core of the cilium, the axoneme, and they protrude into the extracellular space, and have unique mechanisms to tightly control their internal and membrane composition. These features ensure that the cilium is primed to perform highly regulated signaling, mechanosensory [47]. During the establishment and migration of cell morphology, the primary cilium will also be affected. Since the direction of cell migration is related to the establishment of cell polarity, POPX2 phosphatase participates in the necessary pathways for the formation of primary cilium. The overexpression of POPX2 phosphatase will also cause the loss of cell polarity during migration [48]. As a result, the formation of primary cilium is related to the establishment and migration of cells [49]. For example, by controlling CD44-mediated osteopontin signaling and Cdc42-mediated actin cytoskeleton rearrangement, primary cilium can act as osteopontin chemoattractive sensors to regulate the migration of marrow stromal cells (MSCs) [50].

Recent studies have implicated primary cilia have been cited as a potential important mechanosensor in bone cells. Primary cilium are responsible for sensing the mechanical load in bone cells, and bone cells have the function of regulating the expression of primary cilium [51]. Periosteal osteochondroprogenitors (OCPs) directly sense fluid shear and differentiate into bone-forming osteoblasts via their primary cilia. However, this response is essentially lost when the primary cilium is absent [52]. As an extracellular sensor that regulates bone homeostasis, the primary cilium in bones can act as interstitial fluid flow sensors for bone cells and osteoblasts [53]. The establishment of mouse experiments found that mice lacking primary cilium showed severe delayed fracture healing and incomplete fracture healing, and studies confirmed that primary cilium of cells expressing paired related homeobox 1 (Prx1) are necessary for fracture repairment [46]. In the musculoskeletal system, the length of primary cilium is regulated by changes in actin or microtubule networks. The basal body of the cilium is connected to the collar of the foot structure, and the collar extends laterally to form the attachment point of the cytoskeleton microtubules. The microtubules contribute to the structural integrity of the basal body and the anchoring of the primary cilium [54]. Microgravity can eliminate primary cilium and inhibit osteogenesis, and at the same time change the dynamics of the cytoskeleton by causing depolymerization of microtubules in rat calvarial osteoblasts. The depolymerization of microtubules under microgravity conditions is not the cause of the disappearance of primary cilium, and this depolymerization process requires the presence of primary cilium [55]. The abolition of the primary cilia from bone cells attenuates bone formation in microgravity. Reconstruction of the primary cilium may become a potential strategy against bone loss caused by microgravity [56, 57]. The primary cilia senses fluid flow and mediates the mechanical response in osteocytes [58–60]. The primary cilia of MC3T3-E1 abrogation or inhibition attenuates normal osteogenic mechanical response to fluid flow [61–63]. Primary cilia are the sites of Ihh signal transduction in cells, which are essential for the formation of bone and cartilage. Growth plate chondrocytes respond to hydrostatic load by increasing Ihh signaling. This transduction requires the participation of primary cilia [64]. When primary cilia are absent, mechanically induced Ihh signaling in chondrocytes is lost. Chondrocytes are respond compressive forces [65]. In articular cartilage, cilia of chondrocytes respond to compression-induced osmotic changes [66, 67]. Ptch1 and Gli1 expression were increased when growth plate chondrocytes exposed to hydrostatic compression, but this enhanced Indian hedgehog (Ihh) signaling was abrogated which lack primary cilia [64].

## Locomotion induced mechanoresponse of BMSCs

The bone marrow mechanoenvironment is easily changed by physical loading and activities, but it does not mean easy breakage of a balanced bone cell activity. In fact, the loading of whole bone [68] induced increase of bone marrow pressure cannot be easily sensed or transduced by BMSCs [69]. However, loading induced pore deformation of trabeculae could create the marrow flow with the shear stress exceeded the threshold level for BMSCs mechanoresponse [70]. When a cyclic loading is applied on long bones, a shear stress with a magnitude of 1.67 to 24.55 Pa is induced [70] in bone marrow. This could trigger various mechanoresponse of BMSCs including morphological changes, migration, proliferation and differentiation (Table 1).

**Table 1:** Effects of different modes of mechanical loading on BMSC activities

Mode	Cell type	Intensity	Frequency	Duration	Effects on BMSCs	References
Fluid shear stress	Murine MSCs	1/2/5 (Pa)	0.5/1/2 (Hz)	14 (days)	A regime of 2 Pa, 2 Hz, induces the most robust and reliable upregulation in osteogenic gene expression	[71]
	hBMSCs	0.2/> 2 (Pa)	–	3/6/12/24 (h)	Lower shear stress (0.2 Pa) induced hMSC migration through MAPK pathways, whereas greater shear stress (> 2 Pa) hindered cell migration	[72]
	hMSCs	0.1/0.7/2.1/4.2 (Pa)	2.8 (Hz)	30 (min)	Upregulation of bioactive compounds under different magnitudes of fluid shear stress	[73]
Mechanical stretch/tension loading	Mouse BMSCs	3%/8%/13%/18% elongation	0.5 (Hz)	8 (h)	Most significant upregulation of osteogenic markers were observed in BMSCs under 8% strain	[74]
	Rat BMSCs	10% amplitude	1 (Hz)	7/14/21 (days)	Chondrogenic markers were most significantly upregulated at the 21st day	[75]
	Mouse BMSCs	10% elongation	0.5 (Hz)	96 (h)	Osteogenic markers together with Wnt proteins were upregulated	[76]
Compress stress	Rabbit BMSCs	0/90/120/150 (kPa)	–	1 or 6 h/day for 2, 4, or 6 days	Chondrogenic markers were most significantly upregulated under 120 kPa with 1 h/day, which were peak at the 4th day	[77]
	Rat BMSCs	90/120/150/180 (kPa)	–	1 (h)	ANTXR1 together with chondrogenic markers were most signifcantly increased under 120 kPa	[78]
	Rat BMSCs	90 (kPa)	–	1 (h)	Hydrostatic pressure promoted cell cycle initiation and stress fibre assembly	[79]

### Effects of mechanical loading on morphology and migration of BMSCs

Mechanical stimulus induced morphological changes and migration of BMSCs occur prior to proliferation and differentiation [80]. A conformational change occurs immediately after external force applied on cell bodies with the establishment of fibronectin (FN)-integrin-cytoskeletal connection [81]. Just within 90 s, the shear stress from ECM induces the formation of initial adhesion [82] which facilitates the movement of BMSCs [83]. Ultimately, continuous fluid flows in single direction could guide the migration of BMSCs following the flow direction [83]. This is because the first adhesion of cell bodies is always assembled at the leading edge of the moving direction [29]. These adhesive structures could pull the polymerizing actin filaments and transfer the power of retrograde flow into a driving force to propel BMSC movement [84]. Interestingly, a high speed of BMSC migration requires a proper force applied on the cell surface and overloading could even prevents the movement [83]. The release of vinculin induced disruption of mechanosensitive link is probably account for a pause of BMSC migration [84]. Since the lifetime of focal adhesion (FA) is only about 20 min, fast-moving BMSCs cannot maintain their speed when a remodelling of FA occurs [33, 83].

### Effects of mechanical loading on differentiation and proliferation of BMSCs

During the past decades, a number of studies have revealed a close relationship between fluid shear stress and BMSCs activities, especially proliferation and osteogenic differentiation [85, 86]. Notably, many of these studies suggested that both the proliferation and differentiation of BMSCs are dependent on strain magnitudes and loading frequency. In vitro, an upward trend of proliferation of rat BMSCs was observed with the increase of flow rate and peaked at 0.4 mL/min, followed by a downward trend thereafter [86]. Similar trend was observed in the study of human BMSCs where a shear stress of 20 dyn/cm^2^ could trigger 126% increase of proliferation [10]. Considering the predicted fluid shear rates of ambulatory motion to be between 8 and 30 dyn/cm^2^ [87] which strengthens osteoblastic bone formation [88–91], it is reasonable to speculate that locomotion induced shear stress could enhance the BMSCs proliferation prepared to refill the osteoblast population.

The differentiation of BMSCs, on the other hand, requires more strict conditions. It occurs only when the appropriate strain magnitudes, loading frequency and duration are together satisfied [85]. To achieve it, intermittent FSS rather than continuous FSS should be applied to BMSCs with a duration of 7–14 days [85]. This is probably due to the factor that intermittent mechanical stimulation provides cells with quiescent periods for the reestablishment of adhesive contact and reorganization of actin cytoskeleton [91, 92]. In return, these cellular reorganization processes facilitate the sensation of BMSCs [93, 94]. Under the intermittent mechanical stimulation, the increase of osteogenic was reported to begin on day 4 and was highest on day 7. This was followed by the increase of OCN level which marks the anaphase of osteoblast formation [85]. As the activities of BMSCs varies with the change of mechanical stimulation, it is not surprising that BMSCs can differentiate into various cell types under different loading conditions. In vitro, cloned mouse bone marrow mesenchymal stem cells have been directed to differentiate into adipocytes, osteoblast‐like cells, chondrocytes as well as fibroblast-like cells under different condition of mechanical stimulation [9]. In fact, in human body, the multipotency of MSCs is system restricted and cell autonomous [18]. Accordingly, locomotion hardly induces the multiple differentiation of BMSCs, thus, only influences bone tissue development.

### Exercises in regulation of BMSC activities and bone mass

**Fig. 1:**
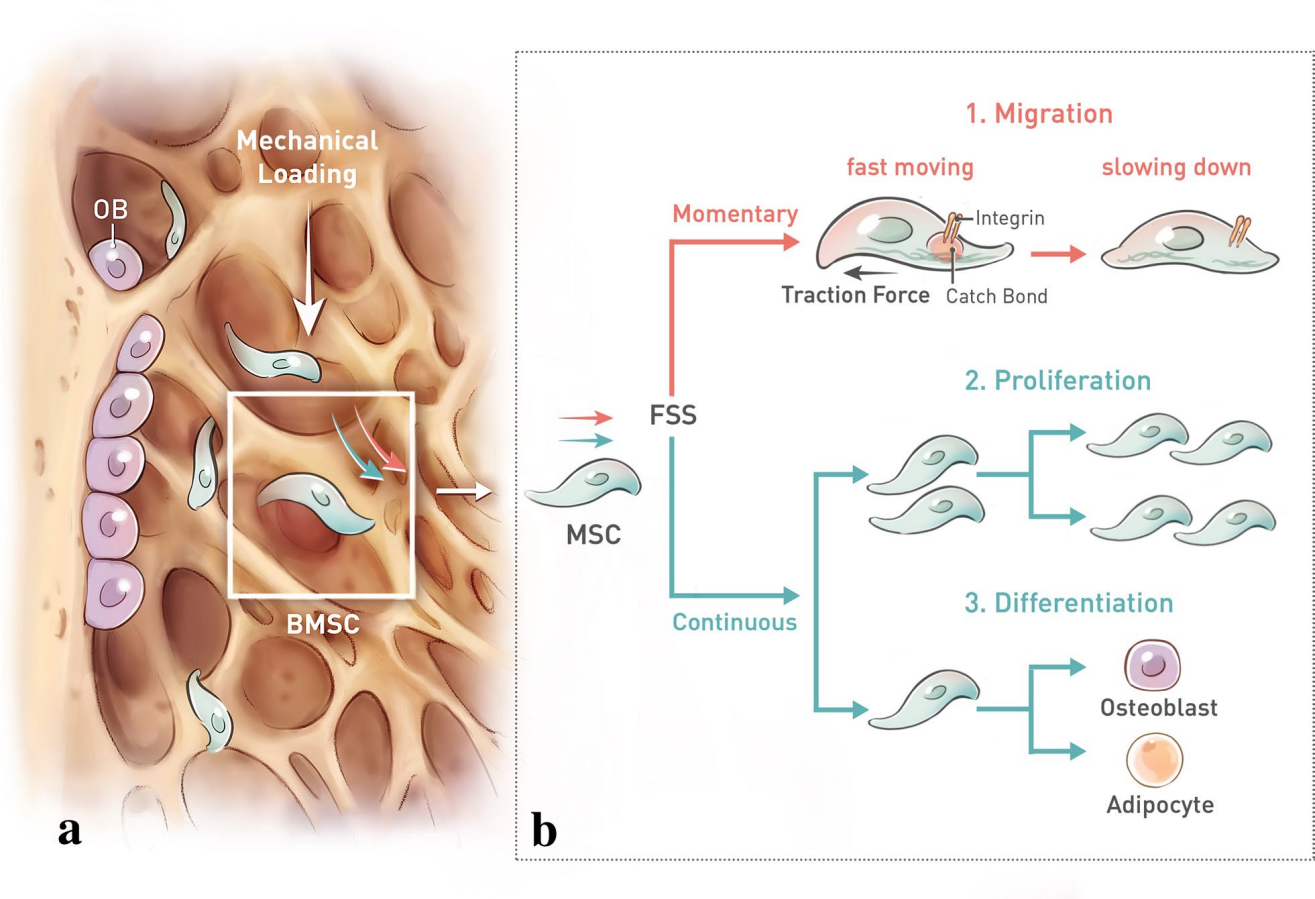
Effects of mechanical loading on BMSCs activities. **a** Mechanical loading, generated by locomotion, from longitudinal direction induces the deformation of trabecular porosity. Deformed trabecular network generates the fluid flow of extracellular matrix (ECM). The fluid shear stress (FSS) subsequently activates the mechanoresponse of BMSCs. **b** FSS majorly induces several cellular activities of BMSCs. First, mechanical signal could easily induce the deformation of BMSCs within 90 s. The change of cell shape is followed by cell migration through ECM-integrin-actin cytoskeleton pathway. During the movement of BMSCs, focal adhesion (FA) is formed to provide the “catch bond” against the intracellular traction force, pulling the cell forward. The disassembly of FA marks the end of movement. Second, continuous mechanical stress could lead to an increase of BMSCs proliferation and differentiation. The direction of BMSCs differentiation depends on the magnitude and frequency of FSS

Considering the effects of mechanical loading on BMSC activities, the regulation of exercise in bone mass is believed to be intensity and duration-dependent (Fig. 1). A recent study reported that only moderate-intensity running could induce increase of bone mass, whereas either low-intensity or high-intensity running had no significant effects on bone formation [95]. In light of this study, our group further investigated the mechanisms of medium-intensity treadmill exercises induced osteogenesis and found out that BMP/Smad signalling pathway played a critical role in osteogenic commitment of BMSCs [96]. In addition to intensity, duration is another factor that determines the effects of exercises on bone formation [97]. Current studies mainly focused on long-term exercises [98]. While it was believed that over 8 weeks of exercises could positively regulate BMSC activities, the effects of short-term exercises remained controversial. In a time course study of jumping exercise, it was reported that there was no significant change of bone response after 2 weeks of jump exercise [99], but 3 weeks or longer period of exercise could increase the bone quality [99]. However, this result was not completely consistent with a subsequent study where C57BL6 male mice were applied to short-term running exercise. In this case, 3-week-duration exercise could not change the size or shape of bone except for an increase of pre-existing bone quality [100]. Nevertheless, both studies showed that short-term exercises may not be effective to bone formation, which is probably due to a shortage of time for the completion of BMSC differentiation.

## Locomotion in relation to signalling pathways for BMSC cell fate (Fig. 2)

**Fig. 2:**
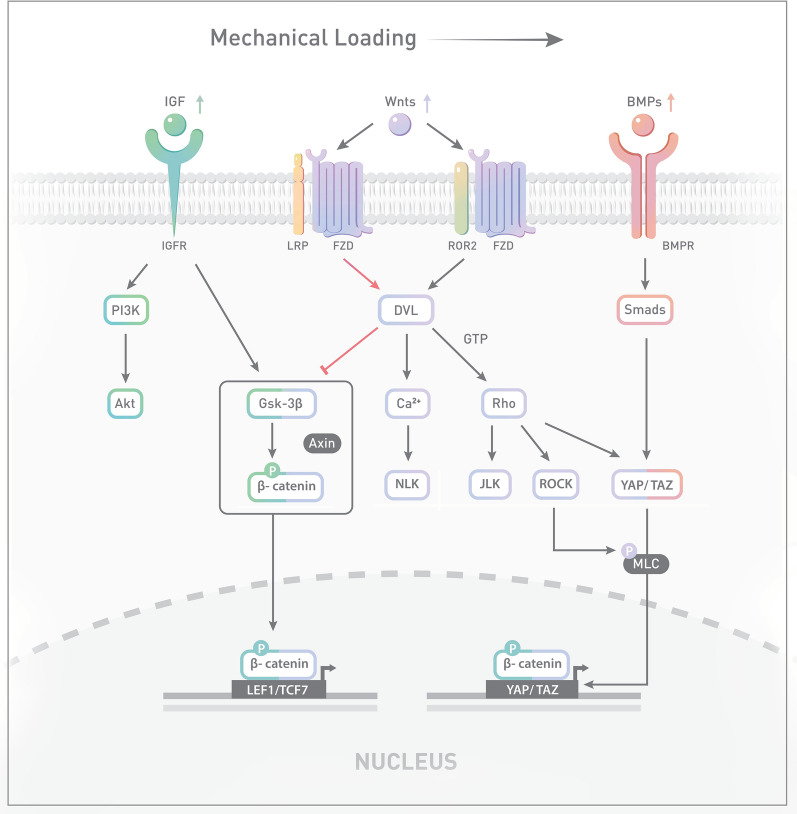
Signalling network of BMSCs under mechanical loading, especially the fluid shear stress (FSS). Mechanical cues could activate several signalling pathways including Wnt, BMP and IGF signalling. Both canonical (red arrowheads) and non-canonical Wnt (black arrowheads) pathways are activated under mechanical loading. In canonical Wnt signalling, DVL inhibits the phosphorylation of β-catenin induced by GSK-3β, which is also downstream to IGF proteins. On the other hand, binding of Wnt ligands to Frizzled and Ror2 co-receptor initiates non-canonical Wnt signalling. It subsequently activates RhoA/Rock signalling which interacts with BMP/Smad signalling through the regulation of YAP/TAZ pathway. Translocation of β-catenin and YAP/TAZ facilitates osteogenic differentiation and migration of BMSCs, respectively

### RhoA/ROCK pathway

The Rho/Rho-associated protein kinase (Rho/ROCK) signalling is important in MSC mechanobiology owing the ability for lineage switch in MSCs [101]. The initiation of chondrogenesis and adipogenesis requires a downregulation of RhoA pathway in MSCs, while the condition to commence an osteogenic differentiation is to the opposite [102, 103]. RhoA/ROCK pathway mediated MSC differentiation has been proved to correlate with a morphological change of MSCs [104]. Such a correlation provides an idea that physical activities could determine the cell fate of differentiation through the regulation of cell shape. Thereby, it is supposed that locomotion in a certain range of magnitude and frequency could activate the RhoA/ROCK casade to promote osteogenesis through morphological regulation of cell body. Of note, the polymerization of cytoskeletal structure normally promotes MSC osteogenic differentiation [104]. This process is often observed in moving cells and static MSCs always lack the potential for osteogenic lineage commitment [105]. As myosin light chain is a downstream target of ROCK, mechanical stimulus induced RhoA/ROCK signalling activation is always accompanied by an increase of actin–myosin contractility [106]. As a result, the increase of cellular tension is normally observed before the commitment of osteogenic differentiation [104]. On the contrary, inhibition of ROCK signalling promotes Chondrogenesis [103] or adipocytogenesis [104]. This normally occurs in soft tissues where the low stiffness of substrates cannot provide enough tension for cytoskeletal reorganization [107].

### IGF pathway

The IGF signalling is mainly comprised of IGF-1, IGF-2 and their type I, type II receptors [108]. In the skeletal response to mechanical loading, IGF-1 was observed to increase in BMSCs [109], osteoblasts [110] and osteocytes [111]. By contrast, skeletal unloading blocks the interaction of IGF-1 to its receptor in BMSCs [109]. As a result, Ras GTPase/MAP kinase (MAPK) and Phosphatidylinositol-3-kinase (PI3K)/Akt signalling, two downstream pathways, were inhibited. This could lead to an inhibition of BMSCs proliferation, since these two pathways are both significant mediators of cell proliferation [112, 113]. On the other hand, a remarkable decrease of osteogenic colony number and periosteal bone formation were observed in unloading condition, which indicates an important role of IGF signalling in loading induced BMSC differentiation [114]. Concomitant with the resistance to IGF-1, downregulation of β1 and β3 integrin subunits were also detected in the absence of loading [115]. This suggests that integrin, as the initial point of cellular mechanosensation [29], is the upstream of IGF signalling. Of note, inhibition of integrin by mechanical unloading specifically disrupt IGF signalling but not other growth factors such as platelet-derived growth factor (PDGF) [115]. To further investigate IGF signalling in BMSCs mechanotransduction, IGF-1Rflox/flox animals were established for the deletion of IGF-1 receptors [116]. In this model, shear stress induced phosphorylation of ERK was significantly reduced compared to the control group with intact IGF-1 receptors [116]. This result suggests that some downstream factors of mechanotransduction in BSMCs are also dependant on IGF signalling.

### BMP pathway

Bone morphogenetic proteins (BMPs) are known as another group of mechanically sensitive cytokines upstream to locomotion induced BMSCs differentiation [117, 118]. Within the BMP family of more than 20 members, BMP-2, -4, -7, -9, and -13 are most frequently analysed being important participators in BMSCs differentiation [13]. Among them, BMP-2 and BMP-4 are suggested to be involved in mechanical stimulus induced osteogenic lineage commitment [119]. Interestingly, the initiation of mechanical loading induced BMP signalling is believed to initiate from integrin-BMP interactions [117]. Since the BMP receptors are colocalized with αvβ integrins [120] and integrin signalling complexes [121, 122], it is supposed that an alteration of BMP receptor endocytosis would occur as a result of loading induced morphological changes of integrin complexes [117]. Following that, the activation of BMP-2 facilitates the phosphorylation of Smad1/5/8, which subsequently regulates the transcript of the genes involved in early osteoblastic differentiation event [117]. In this case, Runx2-Smad interaction is the key for osteogenic differentiation. The mutation of Runx2 fails to induce BMP/Smad-mediated osteoblastic differentiation [123]. Different to BMP-2, BMP-4 is suggested to have different response to mechanical stretch. While no significant changes of Smad1/5/8 occurred, ERK1/2 phosphorylation was observed downstream to BMP-4. This inhibited the adipogenic differentiation by suppressing transcript of PPARγ, C/EBPα and aP2 [124]. In addition to integrin-BMP interaction, concentration of BMP proteins could also determine BMSC cell fate. While concentrated BMP-2 promotes osteogenic differentiation, decrease of BMP concentration facilitates the adipogenic differentiation [125]. Accordingly, although not all BMP members were proved to be upregulated under loading condition, locomotion does have the promotional effects on BMP expression which enhances bone formation.

### Wnt pathway

Wnt signalling is generally subdivided into canonical Wnt and non-canonical pathways characterized by its dependency on β-catenin [126, 127]. Canonical Wnt signalling initiates from the binding of Wnt ligands to Frizzled receptors located on the cell membrane. The activation of Wnt signaling facilitates the accumulation of β-catenin as it inhibits glycogen synthase kinase-3β (GSK-3β) induced β-catenin phosphorylation. As a result, β-catenin is able to be translocated to nucleus for the expression of target genes [128]. By contrast, non-canonical Wnt signalling does not require the involvement of β-catenin and is suggested to be diverse, which includes Wnt/JNK (c-Jun N-terminal kinase), Wnt/Rho and Wnt/calcium signalling pathways [129].

Activation of Wnt signalling is a common response to mechanical loading [130]. Clinically, strength and power training were observed to upregulate the expression of Wnt-related genes in human body [131]. This suggests a high mechanosensitivity of Wnt signalling. In condition of mechanical stimulation, both canonical and non-canonical Wnt pathways are involved in the determination of BMSC cell fate. The investigation of how canonical Wnt affects osteoblastic lineage commitment suggested that mechanical strain induced switch of adipogenic differentiation to osteogenic differentiation was dependent on a preservation of β-catenin [132]. Of note, high-magnitude mechanical stress could inhibit canonical Wnt signalling induced osteoblastic differentiation. In this case, an inhibitory effect of large-magnitude loading on PI3K/Akt pathway results in the accumulation of GSK-3β leading to the phosphorylation of β-catenin [133].

On the other hand, oscillatory fluid flow could also activate non-canonical Wnt signalling [134]. The activation of non-canonical Wnt signalling cascades requires binding of Wnt ligands to Frizzled and Ror2 co-receptor complexes [13]. Overexpression of Orphan Receptor Tyrosine Kinase Ror2 was proved to enhance osteogenic differentiation [135]. The binding of Wnt proteins to their receptors could affect one of the downstream factors RhoA [136], which may lead to enhanced osteogenic differentiation [134].

Although both of canonical and non-canonical Wnt signalling activated by mechanical stimulation exhibit ability for bone formation, the activation of diverse Wnt pathways may not collaboratively enhances osteoblastic differentiation as crosstalk in between Wnt signalling could antagonise the effects on BMSCs differentiation [137]. Therefore, locomotion induced mechanical stimulation may not simply guide Wnt-mediated osteogenic differentiation of BMSCs. A proper magnitude of loading that could activate Wnt signalling while dispel the antagonism between canonical and non-canonical Wnt seems to be the key for promotional effects of Wnt pathways in bone formation.

## How to maintain bone health by understanding locomotion induced BMSCs activities

The effects of locomotion are always the focus in bone-related research. It is believed that there is a close relationship between locomotion and osteogenic lineage commitment. Although many in vitro studies have suggested that BMSCs could sense the fluid flow shear stress and initiate osteogenic differentiation afterwards, how to develop a proper physical activity by understanding the mechanical characteristics of BMSCs remains a mystery. So far, it has been suggested that regular physical activities could enhance proliferation and osteogenic differentiation of BMSCs in vivo [7] and significantly decrease marrow adipose tissue [138]. These results are consistent with our general ideas that locomotion could strengthen our bones. In terms of the correlation between intensity of activities and bone formation, moderate intensity of physical activities seems to be most efficient to enhance bone strength [8]. Accordingly, it dispels the bias that skeletal benefits of physical activities are proportional to load magnitudes [139]. This is consistent with the characteristics of BMSCs that osteogenic lineage commitment requires 7–14 mechanical stimulation [85]. Moreover, if an endurance training was conducted, BMSCs could even promote hematopoiesis in bone marrow [140].

### Relationship between exercises and bone mass

The characteristics of BMSCs provides a guidance of appropriate exercise mode for strengthening our bone. Both clinical trails [141] and animal studies [96] suggested that load-bearing exercises were most effective to increase bone strength. In children and adolescents, whose skeleton was undergoing development, the effects of exercises on bone mass seemed to be most significant [142]. For example, long-term load-bearing activities, such as jumping, were suggested to induce no less than 1% increase of bone mass and approximately 1–8% improvement in bone strength in adolescents [143, 144]. In comparison, the effects of exercises on adults are more of prevention of bone loss rather than increase of bone mass. For instance, only 0.5–2.5% improvement of bone strength was observed in premenapausal women who participate in sustained weight bearing resistance exercises. However, this positive effect on bone is still important in resistance of osteoporosis [145], considering a 0.5–2.5% annual loss of bone mass after menopause [146].

On the other hand, low-impact or non-load-bearing exercises are not osteogenic. For example, over 1% bone mineral density (BMD) loss at the hip and lumbar spine was detected in female cyclists in a 12-month-period study [147]. Similarly, while yoga and swimming were considered to be lifetime fitness activities, they showed little effects on increase of bone mass [148, 149]. These results were consistent with BMSC mechanobiology that activation of BMSC differentiation for extra bone formation purpose was loading-dependent.

In addition to the form of exercises, duration is another factor to strengthen skeleton. Since BMSCs require a period for the completion of differentiation, short-term exercises may not enough for osteogenic commitment. Therefore, long-term exercises are considered to be effective on bone health. For example, it has been reported that a 7-month-periode high-impact training, followed by 7 months of normal activities resulted in a 4% increase of femoral head BMC and area [150]. Similar results could be seen in a group of adolescent girls who participated in a 9-month-period jumping exercises combined with a 20-month follow-up activities, where 6% higher increase in BMC at the lumbar spine was detected compared to the control group [151].

### Application of BMSC mechanical characteristics in translational medicine

The mechanical characteristics of BMSCs also provides possibility for translational medicine. BMSC-based therapy in bone defects healing has been investigated in several previous studies [152–154]. However, these studies only discussed the osteogenic potential of BMSCs in promotion of bone healing without the mention of mechanical stimulation. Although direct BMSC transplantation could enhance bone regeneration, the repair of large bone defects is still not ideal without the application of mechanical loading which is critical to proper endochondral bone development of fracture healing [155]. By using internal fixation plates of variable stiffness, modulated ambulatory load was applied to engineered mesenchymal condensations in a recent study of rat critical-sized bone defects [155]. In this case, modulation of mechanical loading could make a switch of BMSC decision towards either chondrogenesis or angiogenesis [155], thus, contribute to different stages of endochondral bone regeneration. Following this study, the same research group developed scaffold-free mesenchymal condensations which presented morphogen (TGF-β1 and BMP-2), the key factors for the initiation of endochondral bone regeneration. The combination of engineered mesenchymal condensations and mechanical stimulations showed a promising effects in large bone defect healing [156].

In addition to bone tissues, combination of mechanical loading and BMSCs have been applied to other tissues. Considering their chondrogenic potential, BMSCs were recently applied to cartilage regeneration [77]. To maximize the chondrogenic differentiation potential, the seed cells were pressure-pretreated in the study. As a result, boundaryless repair between the neocartilage and residual host cartilage was achieved [77]. Moreover, mechanoresponse of BMSCs could be used in tendon regeneration. A recent investigation reported that combined stimulation of cyclic stretch and TGF-β treatment could promote tenogenic differentiation of BMSCs. The coupled mechano-chemical induction of BMSC-based treatment also showed enhanced tendon regeneration in vivo [157].

Although there is insufficient evidence concerning the efficacy of BMSC mechanoresponse-based treatment in clinical trials, current progress in this area sheds light on a novel approach for tissue regeneration.

## Conclusion

In summary, recent studies have established a strong relationship between locomotion and bone health based on the mechanoresponse of BMSCs. So far, it is well confirmed that constant loading with appropriate intensity could promote the proliferation and osteogenic differentiation of BMSCs [85, 86]. Accordingly, long-term load-bearing exercises were believed to be most effective in maintenance of bone mass at different stages of life [143, 144]. BMSC mechanoresponse also provides promising strategies for large bone defects healing and tissue regeneration [77, 155–157]. Combination of BMSC treatment and biomaterials could be a reliable option for bone tissue healing in the future. Although there is a large progress in understanding of BMSC mechanoresponse recently, technical development for simulation of BMSC mechanical environment is still needed. As the in vitro studies cannot reflect the real mechanical stimulus that BMSCs sense in vivo, the threshold level of exercises for bone formation is still unanswered. Besides, as bone fracture healing usually require Immobilization, how to develop the application of BMSC mechanoresponse in bone tissue healing without affecting the stabilization of fracture site needs to be further investigated.

## Data Availability

Not applicable.

## Abbreviations

BMP: Bone morphogenetic protein
IGF: Insulin-like growth factor
Hh: Hedgehog
BMSCs: Bone marrow mesenchymal stem cells
ECM: Extracellular matrix
FA: Focal adhesion
FSS: Fluid shear stress
OFF: Oscillatory fluid flow
GSK-3β: Glycogen synthase kinase-3β
IMP: Intramedullary pressure
PPARγ: Peroxisome proliferator-activated receptor γ
Runx2: Runt‐related transcription factor 2
TAZ: Transcriptional co-activator with PDZ-binding motif
YAP: Yes-associated protein
GPCRs: G protein-coupled receptors
MCHR1: Melanin-concentrating hormone receptor 1
OCPs: Osteochondroprogenitors
Prx1: Paired related homeobox 1
Ihh: Indian hedgehog
FN: Fibronectin
FA: Focal adhesion
ROCK: Rho-associated protein kinase
MAPK: MAP kinase
PI3K: Phosphatidylinositol-3-kinase
JNK: C-Jun N-terminal kinase
BMD: Bone mineral density
